# Investigation of AlGaN/GaN HFET and VO_2_ Thin Film Based Deflection Transducers Embedded in GaN Microcantilevers

**DOI:** 10.3390/mi11090875

**Published:** 2020-09-20

**Authors:** Ferhat Bayram, Durga Gajula, Digangana Khan, Goutam Koley

**Affiliations:** 1 Holcombe Department of Electrical and Computer Engineering, Clemson University, Clemson, SC 29634, USA; digangk@clemson.edu (D.K.); gkoley@clemson.edu (G.K.); 2 School of Electrical and Computer Engineering Georgia Institute of Technology, Atlanta, GA 30332, USA; gdraophy@gmail.com

**Keywords:** microcantilever, VO_2_, metal insulator transition (MIT), AlGaN/GaN, heterojunction field effect transistors (HFET), deflection transducer, strain sensor, MEMS

## Abstract

The static and dynamic deflection transducing performances of piezotransistive AlGaN/GaN heterojunction field effect transistors (HFET) and piezoresistive VO_2_ thin films, fabricated on GaN microcantilevers of similar dimensions, were investigated. Deflection sensitivities were tuned with the gate bias and operating temperature for embedded AlGaN/GaN HFET and VO_2_ thin film transducers, respectively. The GaN microcantilevers were excited with a piezoactuator in their linear and nonlinear oscillation regions of the fundamental oscillatory mode. In the linear regime, the maximum deflection sensitivity of piezotransistive AlGaN/GaN HFET reached up to a 0.5% change in applied drain voltage, while the responsivity of the piezoresistive VO_2_ thin film based deflection transducer reached a maximum value of 0.36% change in applied drain current. The effects of the gate bias and the operation temperature on nonlinear behaviors of the microcantilevers were also experimentally examined. Static deflection sensitivity measurements demonstrated a large change of 16% in drain-source resistance of the AlGaN/GaN HFET, and a similarly high 11% change in drain-source resistance in the VO_2_ thin film, corresponding to a 10 μm downward step bending of the cantilever free end.

## 1. Introduction

Micro and nanoelectromechanical systems (M/NEMS) have been one of the major research areas spanning several decades due to many of their attractive attributes, including scalability, integration capability, reliability, and design variety [1,2]. Among M/NEMS devices, micro and nanocantilevers have been extensively studied, especially after their sensitivity and application potential was demonstrated by atomic force microscopy [3,4,5,6]. Besides material characterization applications, these structures, resembling tiny diving boards, have been incorporated into physical, chemical, and biological sensing applications, due to their ultra-high sensitivity to physical property changes [7,8,9]. The sensing operations with microcantilevers are based on tracking static or dynamic tip deflections, measured traditionally using optical read-out technologies. Even though optical deflection measurement offers very high resolution, excellent sensitivity, and low noise, it is not practical for an array of micro-resonators requiring simultaneous deflection measurements, as it would be immensely bulky and expensive [10]. In order to overcome the challenges associated with the traditional optical based detection methods, integrated photonic-based waveguide and optical cavity systems have been proposed [11,12,13,14]. Besides optomechanical deflection transducing techniques, recent research has focused on developing highly sensitive piezoresistive and piezotransistive strain sensing structures fabricated on cantilevers, by taking advantage of semiconductor micromachining techniques.

Microcantilevers with integrated piezoresistors at their base, measuring the tip deflections through resistance changes due to applied strain, have been extensively fabricated using Si substrates [4,6,15]. An applied strain originating from the cantilever tip deflections causes changes in carrier mobility, which results in resistance alterations in the Si piezoresistive displacement transducer [16]. However, piezoresistive Si based microcantilevers suffers from high thermal/electrical noise, and low displacement resolutions and sensitivities [10]. As an alternative to these Si based strain transducers, piezotransistive AlGaN/GaN heterojunction field effect transistors (HFET), embedded on GaN microcantilevers as the deflection transducer element, have demonstrated superior deflection sensitivities compared to the Si piezoresistive cantilevers [17,18,19,20]. Taking advantage of the strong piezoelectric properties of the AlGaN/GaN heterostructure, higher deflection sensitivities were achieved since an externally applied strain not only changes the carrier mobility, but also modulates the density of the two-dimensional electron gas (2DEG) formed at the AlGaN/GaN interface [18]. In addition to the piezoelectric properties, the introduction of a gate structure to externally control the 2DEG density in the interface, can help with tuning the gauge factor and deflection sensitivity to very high values, more than 100 times that of the Si piezoresistors [18,21].

As another candidate to overcome the sensitivity limitations of Si based piezoresistive displacement transducers, vanadium oxide (VO_2_) thin films have gained great attention due to unique metal insulator transition (MIT) properties [22,23,24,25,26]. Near temperatures of 68 °C, the initial monoclinic insulating structure of VO_2_ transforms into a rutile metallic phase. During this phase transition, the optical and electrical properties of VO_2_ change abruptly. Besides temperature, insulator metal transition of VO_2_ can be triggered with external physical parameters, including electrical field and applied strain. Variation in externally induced strain, in particular, can result in significant variation of the VO_2_ resistance [27,28,29,30]. Therefore, VO_2_ is another attractive alternative to Si piezoresistors that is suitable for applications requiring strain sensing.

In this work, the transducer performances of AlGaN/GaN HFET and VO_2_ thin film embedded at the base of GaN microcantilevers, operated in linear and nonlinear dynamic modes and static modes, have been investigated. Although we have previously investigated GaN microcantilevers with integrated AlGaN/GaN HFET and VO_2_ thin film deflection sensors separately, [18,26] a direct comparison of the transducer performance under the same strain conditions was not performed. In addition, tunability of the sensitivity of the VO_2_ sensor and its non-linear modes were not investigated. Herein, we fabricated VO_2_ thin film (thickness of 70 nm) and AlGaN/GaN HFET deflection transducer (with the same nominal dimension of 35 × 35 μm) integrated GaN microcantilevers with the same length and width dimensions of 250 × 100 μm. While standard photolithographic fabrication methods were employed to form the AlGaN/GaN HFETs, the VO_2_ thin film transducers were grown using a low-pressure chemical vapor deposition system [17,18,26,31,32]. The microcantilevers were excited with the same piezoactuator attached underneath the microcantilevers to ensure similar test conditions. The deflection sensitivities of the AlGaN/GaN HFET and VO_2_ thin film-based transducers were found to be strongly dependent on the gate bias and temperature, respectively. In both linear and nonlinear dynamic operational regimes, piezoresistive VO_2_ thin film demonstrated a comparable displacement transduction performance to the AlGaN/GaN HFET transducer. Static deflection experiments also demonstrated very similar deflection sensitivities for the AlGaN/GaN HFET and VO_2_ thin film transducers. Additionally, the effects of temperature on the intrinsic nonlinearities of piezoresistive VO_2_ thin film embedded cantilever were studied.

## 2. Materials and Methods

**Figure 1:**
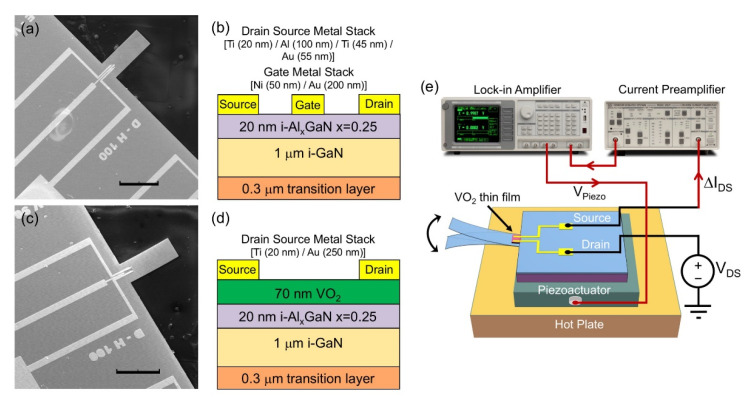
(**a**) Scanning electron microscope (SEM) image of an AlGaN/GaN heterojunction field effect transistor (HFET) embedded GaN microcantilever with dimensions of 100 × 250 μm. Scale bar is 200 μm. (**b**) Layer structure of the fabricated AlGaN/GaN HFET. The transition and i-GaN layers form the thickness of the microcantilevers. (**c**) SEM image of VO_2_ thin film embedded GaN microcantilever with dimensions of 100 × 250 μm. Scale bar is 200 μm. (**d**) Layer structure of the VO_2_ thin film embedded microcantilever. Similarly to the AlGaN/GaN HFET embedded microcantilever, the transition and i-GaN layers form the microcantilever thickness. (**e**) Schematic of the experimental setup utilized to characterize the VO_2_ thin film based GaN microcantilever.

GaN microcantilevers with integrated AlGaN/GaN HFETs and piezoresistive VO_2_ thin films were fabricated using epitaxial layers of III-Nitrides, consisting of 20 nm Al_x_GaN (x = 0.25), 1 μm GaN, and 0.3 μm buffer layers grown on a 0.6 mm Si (111) substrate. The fabrication process of cantilevers with integrated AlGaN/GaN HFET has been discussed in detail elsewhere [17,18,33,34]. Briefly, the top AlGaN layer was etched using an inductively coupled plasma tool to define the HFET, and the VO_2_ mesa regions with dimensions of 35 × 35 μm. Then, the GaN layer was etched accordingly to shape the microcantilever outline. For AlGaN/GaN HFET microcantilevers, the metal stack of Ti (20 nm)/Al (100 nm)/Ti (45 nm)/Au (55 nm) was deposited using an e-beam evaporator to form the HFET ohmic contacts. Finally, Schottky-metal contacts of Ni (50 nm)/Au (200 nm) and the probe metal stack of Ti (20 nm)/Au (150 nm) were deposited. For VO_2_ thin film embedded GaN cantilevers, a 70 nm vanadium layer was deposited on top of the defined area. The deposited thin film was oxidized at 475 °C for 30 min using a home-built low-pressure chemical vapor deposition system [26,31]. Metal stacks of Ti (20 nm)/Au (250 nm) were deposited for establishing ohmic contacts to the VO_2_ thin film. For both AlGaN/GaN HFETs and VO_2_ thin films, probe metal depositions of Ti (20 nm) and Au (250 nm) were employed for wire-bonding to chip carriers. Finally, the Si substrate layer was etched with the Bosch process to release all the microcantilevers. Scanning electron microscope (SEM) images of the fabricated AlGaN/GaN HFET and VO_2_ thin film embedded GaN microcantilevers, with the same nominal dimensions of 100 × 250 μm (width × length) are presented in Figure 1a,c, respectively. Figure 1b,d demonstrate the layer structure of fabricated AlGaN/GaN HFET and VO_2_ films.

**Figure 2:**
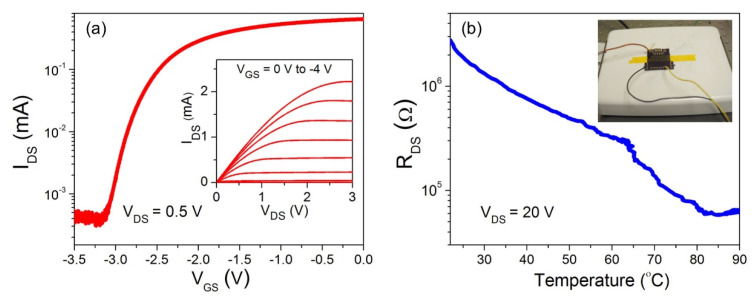
(**a**) *I*_DS_-*V*_GS_ characteristics of the AlGaN/GaN HFET. The inset shows *I*_DS_-*V*_DS_ characterization results. (**b**) Temperature effects on the VO_2_ thin film resistance. A photo of the temperature characterization setup, with the hot plate, the biasing signal lines and the thermo-couple, is shown in the inset.

Figure 1e demonstrates the schematic of the experimental setup used to characterize the VO_2_ transducer embedded GaN microcantilever. The fabricated chips containing AlGaN/GaN HFET and VO_2_ thin film embedded cantilevers were attached (using a high temperature compatible epoxy) to a custom designed printed circuit board (PCB), to easily form electrical connections. A piezo actuator with dimensions of 5 × 5 × 2 mm (Model: PL055.3x from Physik Instrumente GmbH & Co., Karlsruhe, Germany) was attached under the PCB to actuate the microcantilevers. The cantilevers were placed on a hot plate as shown in the schematic. The same piezoactuator was employed in all characterization measurements of the AlGaN/GaN HFET and VO_2_ thin film embedded GaN microcantilevers, to facilitate direct performance comparison between the two different transducers. At first, transistor and metal insulator transition characteristics were investigated for the embedded AlGaN/GaN HFET and VO_2_ thin film, respectively. Figure 2a displays changes in AlGaN/GaN HFET drain-source current (*I*_DS_), with the gate-source voltage (*V*_GS_) at a constant drain-source voltage (*V*_DS_) of 0.5 V. Drain currents below μA level in the cut-off region (*V*_GS_ < −3 V) indicate excellent gate control. The inset of Figure 2a shows typical I-V characteristics of the AlGaN/GaN HFET. The effects of temperature on VO_2_ thin film drain source resistance (*R*_DS_) at a constant *V*_DS_ of 20 V, applied using a source measurement unit (SMU) (B2902A Keysight Technologies Inc., Santa Rosa, CA, USA), are shown in Figure 2b. A photo of the VO_2_ thin film embedded microcantilever on a hot plate, for temperature characterization, is shown in inset of Figure 2b. While the room temperature resistance of VO_2_ thin film was found to be around ~3 MΩ, the resistance reduced down to ~60 kΩ at 80 °C. As the VO_2_ thin film temperature reached the MIT temperature (which is typically found to be slightly above 60 °C for our films), the rate of decline in the R_DS_ with temperature became sharper, as expected. We did not find the change to be very sharp, as observed for large area films on sapphire or SiO_2_/Si [35,36], possibly due to polycrystallinity and defects on the small area deposited VO_2_ films.

After characterizations of the AlGaN/GaN HFET and the VO_2_ thin film, we investigated deflection transduction properties of these transducers embedded at the base of the GaN.

The microcantilevers were operated in their dynamic oscillation mode. The AlGaN/GaN HFET embedded cantilever was excited by the piezoactuator under various alternating current (AC) biases, while a constant *I*_DS_ of 200 μA was applied to the HFET drain from the SMU. The dynamic changes in the *V*_DS_ (∆*V*_DS_), due to piezoactuator-based oscillations of the microcantilever, were measured using a lock-in amplifier (SR850 Stanford Research Systems, Sunnyvale, CA, USA), which was also used to bias the piezoactuator with a variable frequency sinusoidal AC voltage. Detailed experimental procedures of GaN microcantilever dynamic characterizations have been reported elsewhere [18,19,20,33,34]. To characterize the VO_2_ thin film embedded GaN microcantilever, a constant drain source voltage (*V*_DS_) of 20 V was applied to the drain contact of the VO_2_ thin film using the SMU. To measure alterations in the drain source current, *I*_DS_, due to the cantilever oscillation, the source contact was connected to a current pre-amplifier (SR570 Stanford Research Systems) to amplify the current readings. The amplified signal was fed to a lock-in amplifier that measured the AC changes in *I*_DS_. All the measurements were done on top of a hot plate, which enabled the temperature of the VO_2_ thin film to be controlled during the characterization experiments. The temperature measurements were made using a standard thermocouple (k type) and data-acquisition equipment (34972A from Agilent Technologies Inc., Santa Clara, CA, USA).

In addition to dynamic characterization experiments, the static deflection transducing performances of the AlGaN/GaN HFET and the VO_2_ thin film were measured by deflecting the microcantilevers’ free ends by 10 μm downward. A tungsten needle with a tip radius of 7 μm (72T-J3/70 Creative Devices Inc., Middletown, DE, USA) was attached to a computer-controlled nanopositioner (P-611.Z Physik Instrumente GmbH & Co., Karlsruhe, Germany) for the bending experiments. The same constant biases of *I*_DS_ = 200 μA and *V*_DS_ = 20 V used in the dynamic resonance measurements were also applied to the HFET and VO_2_ thin film (using the SMU), respectively. The changes in the V_DS_ of the HFET and the I_DS_ of the VO_2_ thin film, due to the 10 μm downward bending of the microcantilever tip, were recorded.

## 3. Results and Discussion

**Figure 3:**
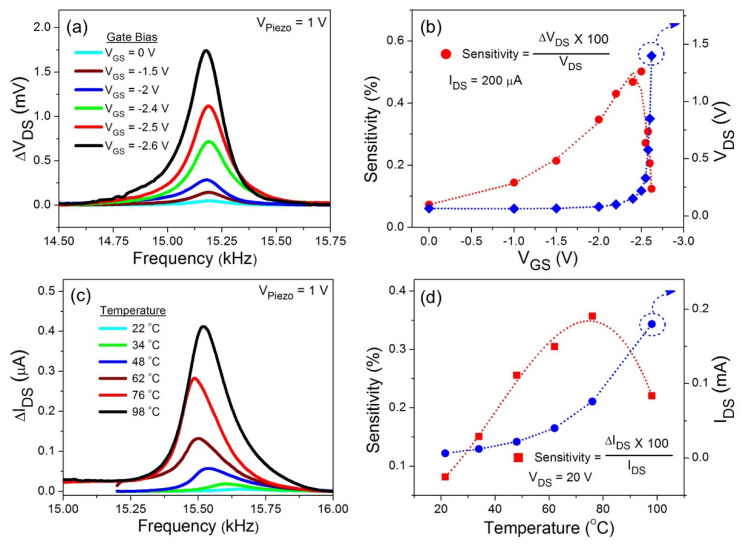
(**a**) Dynamic resonance characteristics of AlGaN/GaN HFET embedded GaN microcantilevers at various gate biases. An AC signal of 1 V was applied to the piezoactuator. (**b**) Calculation of the AlGaN/GaN HFET sensitivity changing with the gate bias, as shown in red circles. The HFET V_DS_ variation due to the gate bias is shown in the right axis. (**c**) Effects of the temperature on the dynamic resonance behavior of the VO_2_ thin film embedded GaN microcantilever, while the piezoactuator was biased at 1 V. (**d**) VO_2_ thin film deflection sensitivity calculated at various temperatures. As the temperature increases, resistance of the VO_2_ thin film reduces. Therefore, at a constant *V*_DS_ of 20 V, I_DS_ increases with temperature, as shown in the right axis.

We have demonstrated in our past studies that the deflection responsivity of the AlGaN/GaN HFET can be manipulated using the gate voltage [17,19,21,34]. On the other hand, we expected the sensitivity of the VO_2_ thin film deflection transducer to also be tunable based on the temperature of the film. To compare the deflection sensitivity of the AlGaN/GaN HFET and VO_2_ thin film in the microcantilevers’ linear dynamic regime, the piezoactuator under the microcantilevers was biased at a constant AC voltage of 1 V. Figure 3a displays the experimental resonance characteristics of the AlGaN/GaN HFET embedded GaN microcantilever at various gate voltages, ranging from 0 to −2.6 V. The resonance frequency (*f*_0_) and the quality factor (*Q*_f_) of the HFET embedded microcantilever were determined from the measurements as 15.150 kHz and 80, respectively. As the channel resistance (*R*_DS_) increased due to higher gate biases, for a constant *I*_DS_ of 200 μA, the ∆*V*_DS_ corresponding to the mechanical oscillations of the microcantilever (at 1 V piezo excitation) also increased. Deflection sensitivity of the AlGaN/GaN HFET transducer at different gate voltages can be calculated using $Sensitivity\;\left(\%\right)=\;\frac{\Delta V_{\mathrm{DS}}\;\times \;100}{V_{DS}}$, where V_DS_ is the drain source voltage at a particular gate voltage, and ∆*V*_DS_ is the HFET resonance amplitude. As shown in Figure 3b, the deflection sensitivity of AlGaN/GaN HFET reaches its maximum point of a ~0.50% change in *V*_DS_, corresponding to a gate voltage of −2.5 V. The sensitivity reduced dramatically at gate voltages higher than this critical bias, even though *V*_DS_ kept increasing, as shown in the right axis of Figure 3b.

The deflection responsivity of the VO_2_ thin film deposited on the GaN microcantilever was also characterized at *V*_Piezo_ = 1 V, at different measurement temperatures. Figure 3c demonstrates the resonance characteristics of the microcantilever with the VO_2_ piezoresistive transducer. The *f*_0_ and *Q*_f_ of the VO_2_ thin film embedded GaN microcantilever were measured as 15.660 kHz and 85 at room temperature, respectively. At a constant *V*_DS_ = 20 V, I_DS_ was modified as the temperature changed, therefore the sinusoidal change in I_DS_ (∆*I*_DS_), captured with the lock-in amplifier (proportional to the amplitude) at the microcantilever resonance, was also altered. As shown in right axis of Figure 3d, the I_DS_ of the VO_2_ thin film increased with the rise in temperature at constant *V*_DS_ as *R*_DS_ reduces. This increase in *I*_DS_ resulted in a higher resonance amplitude. The deflection sensitivity calculated using the formula: $Sensitivity\;\left(\%\right)=\;\frac{\Delta I_{DS}\;\times \;100}{I_{DS}}$, reached ~0.36% in the range of 65–75 °C, where the metal insulator transition of the VO_2_ thin film takes place. Increasing the temperature beyond the critical MIT temperature reduces the displacement sensitivity of VO_2_ thin film, as clearly evident from Figure 3d. We noted that the resonance frequency of the VO_2_ thin film deposited cantilever shifted significantly to lower frequencies as the temperature increased. Reduction in elastic modulus, which directly determines the spring constant of the cantilever, due to an increase in temperature, was a major reason for the observed red shift in resonance frequency [37].

**Figure 4:**
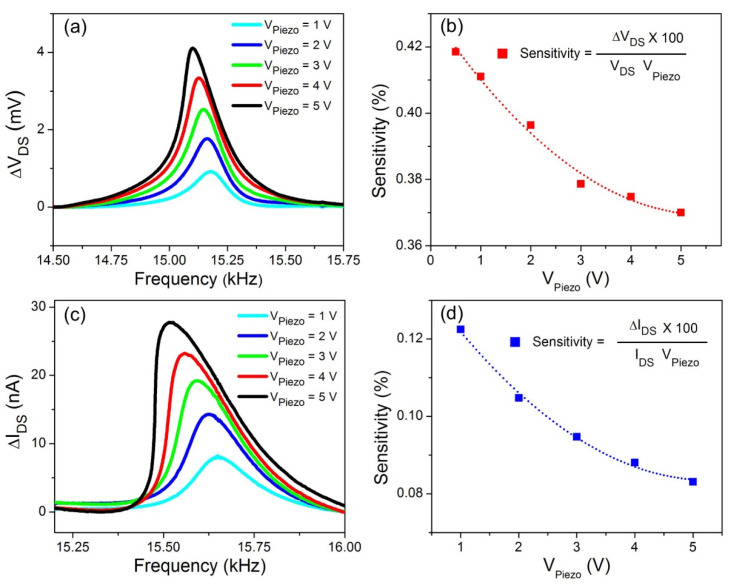
Effects of the excitation amplitude on resonance curves at room temperature and pressure. (**a**) Dynamic resonance characteristics of AlGaN/GaN HFET embedded GaN microcantilevers, at various piezoactuator biases. The resonance frequency of the cantilever shifts to lower frequencies as the excitation signal amplitude increases, due to dominant softening nonlinearities. (**b**) Changes in the AlGaN/GaN HFET sensitivity monotonically reduces as the applied piezoactuator bias is increased. (**c**) Effects of piezoactuator bias on the resonance characteristics of the VO_2_ thin film deposited GaN microcantilever. Similar to the AlGaN/GaN HFET embedded cantilever, softening nonlinearity also reduces the resonance frequency. (**d**) Sensitivity of VO_2_ thin film based displacement transducer reduces as the piezoactuator bias increases, similar to the HFET embedded cantilever.

We also investigated the sensitivities of the AlGaN/GaN HFET and VO_2_ thin film deflection transducers at various piezoactuator biases ranging from 1 V to 5 V, which shifted the resonance mode of the microcantilevers from the linear to non-linear regime. The non-linear operation is particularly interesting due to its wide potential applications in designing ultra-high sensitivity sensors [38,39]. Figure 4a shows resonance curves of the GaN microcantilever with the AlGaN/GaN HFET transducer biased at *I*_DS_ = 200 mA and *V*_GS_ = −2.5 V. Increasing the drive amplitudes (excited by the piezo chip) revealed Duffing type intrinsic nonlinearities of the microcantilever in the fundamental resonance mode. Softening type nonlinearity, shifting the resonance frequency to lower values, was found to be dominant in the first mode, as seen in Figure 4a. According to our previous studies, GaN microcantilevers with widths greater than 70 μm and a length of 250 μm exhibit softening type nonlinearities in their first resonance modes [34]. Therefore, the AlGaN/GaN HFET embedded GaN microcantilever with dimensions of 100 × 250 μm utilized in this study could be expected to manifest softening type nonlinearities at high deflection amplitudes, which was indeed observed in the present study.

The tip oscillation amplitude (*x*_0_) at the resonance frequency of ω_0_ is given by $x_{0}^{2}={\left(\frac{F_{\mathrm{Piezo}}Q_{f}}{k_{\mathrm{eff}}}\right)}^{2}$ where F_Piezo_ is the external effective force applied by the piezoactuator, and *k*_eff_ is the effective spring constant, given as $k_{\mathrm{eff}}=m\omega_{0}^{2}$ (m is the effective mass). The external force applied by the piezo actuator is defined as *F*_Piezo_ = *m*ω_0_^2^*V*_Piezo_*G*_Piezo_ where *V*_Piezo_ and *G*_Piezo_ are the applied piezo bias and the constant piezo displacement coefficient, respectively [34]. Therefore, substituting the piezo force into the deflection equation, we get $x_{0}=V_{\mathrm{Piezo}}G_{\mathrm{Piezo}}Q_{f}$. The amplitude of the tip deflections at the resonance frequency is independent of the intrinsic nonlinearities, including geometric and inertial nonlinearities [40,41]. Since the cantilever tip deflections are directly proportional to the applied piezo bias, *V*_Piezo_ was included in the sensitivity equation. The AlGaN/GaN HFET deflection sensitivity at high oscillation amplitudes was calculated using the formula: $Sensitivity\;\left(\%\right)=\;\frac{\Delta V_{DS}\;\times \;100}{V_{DS}}\frac{1\;V}{V_{\mathrm{Piezo}}\left(V\right)}$. Figure 4b shows changes in sensitivity of the AlGaN/GaN HFET at different excitation biases. The sensitivity was 0.42% at *V*_Piezo_ = 1 V. Increasing the piezo biases gradually reduced the deflection sensitivity of the HFET transducer. At *V*_Piezo_ = 5 V, the sensitivity decreased to 0.37%.

In addition to the AlGaN/GaN HFET deflection characteristics at high oscillation amplitudes, resonance curves of the VO_2_ thin film embedded GaN cantilever at room temperature are shown in Figure 4c. Similarly to the HFET cantilever, the VO_2_ cantilever with the same dimensions features similar softening type nonlinearities in the first resonance mode. The deflection sensitivity of VO_2_ thin film calculated using $Sensitivity\;\left(\%\right)=\;\frac{\Delta I_{DS}\;\times \;100}{I_{DS}}\frac{1\;V}{V_{\mathrm{Piezo}}}$ at various *V*_Piezo_ voltages is shown in Figure 4d. The responsivity of the piezoresistive VO_2_ deflection sensor also reduced monotonically, like the HFET embedded cantilever, as the piezoactuator bias increased. While the resonance amplitude at *V*_Piezo_ = 1 V was measured as a 0.12% change in *I*_DS_ at a constant *V*_DS_ of 20 V, only a 0.08% change in *I*_DS_ was produced due to cantilever oscillations at *V*_Piezo_ = 5 V. These reductions in the sensitivities of piezotransistive AlGaN/GaN HFET and piezoresistive VO_2_ thin film deflection transducers at high drive amplitudes might arise from other factors such as nonlinear damping, which can reduce the quality factor of the resonator at higher amplitudes, thus reducing the normalized resonance amplitude as the applied external force increases [40,41,42].

**Figure 5:**
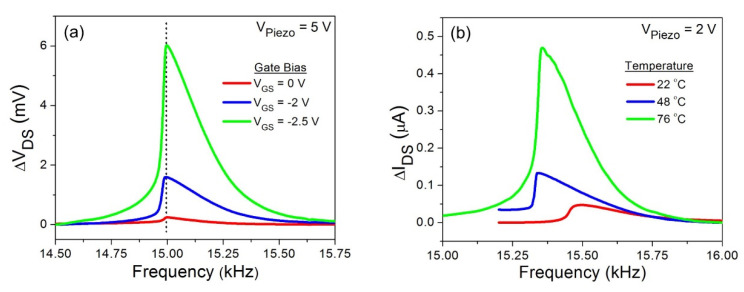
(**a**) Effects of different gate biases on the cantilever resonance in the softening-dominant nonlinear regime. Increasing the gate bias, which alters the HFET sensitivity, does not change the cantilever nonlinear response. (**b**) Temperature influence on the nonlinear response of VO_2_ thin film embedded microcantilever excited at *V*_Piezo_ = 2 V. Temperature not only modifies the sensitivity of the VO_2_ thin film, but also changes the nonlinear behavior of the cantilever.

As mentioned earlier, the gate bias can be used to tune the sensitivity of the HFET deflection transducer. Figure 5a demonstrates the gate bias effects on the resonance curves the of AlGaN/GaN HFET embedded GaN microcantilever, excited in the nonlinear regime at *V*_Piezo_ = 5 V. Backward frequency sweep setting was utilized in all nonlinear curves, since there is a hysteresis between forward and backward curves. For resonators demonstrating softening type nonlinearities, only backward sweep direction exposes a total shift in the resonance frequency [40]. On the other hand, forward frequency sweep needs to be used to identify the frequency shift in hardening dominated nonlinear curves, where the resonance frequency moves to higher frequencies. Higher gate biases result in the enhancement of the HFET resonance amplitude as the sensitivity of the AlGaN/GaN HFET increases. However, the drop frequency, which is a significant feature of these Duffing type nonlinear behaviors, remains stable at 15.00 kHz, as displayed by the dotted line in Figure 5a. The results indicate that the gate bias modifying sensitivity of the HFET based deflection transducer does not affect nonlinear characteristics of the GaN microcantilever. On the other hand, the operating temperature of the piezoresistive VO_2_ thin film transducer can be changed to tune its deflection sensitivity.

Such changes in the operating temperature of the VO_2_ thin film also caused alterations in nonlinear resonance curves as shown in Figure 5b, which was not observed for the HFET embedded cantilever. Even though the sensitivity of the VO_2_ thin film increased towards the critical MIT temperature, the cantilever resonance frequency also shifted to lower frequencies, since utilizing the hot plate to control the VO_2_ thin film temperature also regulated the cantilever temperature. The temperature dependence of elastic modulus, which resulted in red shift of the resonance frequency for linear regime oscillations, as noted in our above discussions, can also change the non-linear behavior of the resonators [37]. In addition, dimensional changes of the cantilever due to temperature rise can modify the intrinsic nonlinearities [34]. A combination of these factors likely resulted in the shift in the drop frequency of oscillations in the nonlinear region, as observed in Figure 5b.

**Figure 6:**
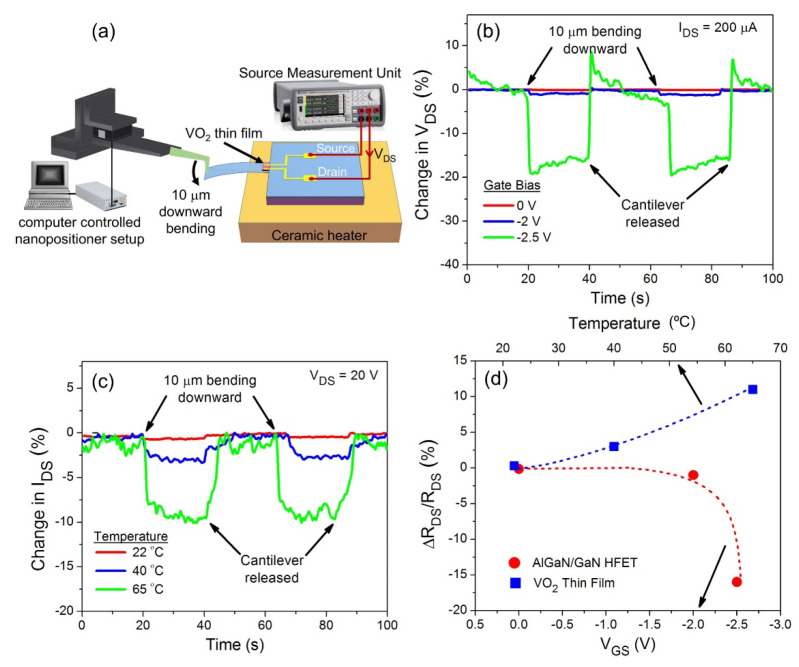
(**a**) Schematic of the experimental setup used to characterize the VO_2_ thin film coated GaN microcantilever through step bending of the cantilever apex. (**b**) 10 μm step bending results of AlGaN/GaN HFET embedded GaN microcantilevers at selected gate voltages. The HFET was biased at *I*_DS_ = 200 μA during downward bending experiments. (**c**) 10 μm downward static bending responses of VO2 thin film, under an applied bias of *V*_DS_ = 20 V, at various temperatures. (**d**) Calculated resistance changes of AlGaN/GaN HFET and VO2 thin film deflection transducers in response to 10 μm downward bending at selected gate biases and temperatures, respectively.

In Figure 6, the static bending experiment results of the AlGaN/GaN HFET and VO_2_ thin film deflection transducers are presented. A schematic of the experimental setup used for the VO_2_ thin film embedded GaN microcantilever is presented in Figure 6a. When the tip of the GaN microcantilever was bent 10 μm downward, the *V*_DS_ of the HFET, under a constant bias of *I*_DS_ = 200 μA and *V*_GS_ = 0 V, was reduced by 0.15%. As shown in Figure 6b, increasing the deflection sensitivity of the AlGaN/GaN HFET, by tuning the gate bias, yielded 1% and 16% reductions in the *V*_DS_ at gate biases of *V*_GS_ = −2 V and *V*_GS_ = −2.5 V, respectively. As the cantilever bends downward, effective tensile stress increases the 2DEG density of the AlGaN/GaN HFET, leading a reduction in the channel resistance of *R*_DS_ [18]. At a constant *I*_DS_, the HFET channel voltage of *V*_DS_ also decreases by a percentage directly proportional to the *R*_DS_ reduction, as the cantilever undergoes a downward bending. Figure 6c demonstrates changes in *I*_DS_ of the VO_2_ thin film under an applied drain bias of *V*_DS_ = 20 V in response to 10 μm downward step bending at various temperatures. While the *I*_DS_ of VO_2_ thin film reduces 0.3% at a room temperature of 22 °C, increasing the temperature towards the MIT temperature enhances the static deflection sensitivity of VO_2_ thin film. As shown in Figure 6c, 10 μm downward step bending yielded 3% and 10% decreases in the *I*_DS_ of the VO_2_ thin film transducer at temperatures of 40 °C and 65 °C, respectively. At a constant *V*_DS_, reduction in the *I*_DS_ due to downward bending corresponds to an increase in the *R*_DS_. This behavior was expected since the resistance of VO_2_ thin film increases due to band structure changes when the VO_2_ thin film is subjected to a tensile stress [43]. Figure 6d displays the sensing results in Figure 6b,c in terms of normalized *R*_DS_ changes, (∆*R*_DS_/*R*_DS_) in the AlGaN/GaN HFET (red circles), and VO_2_ thin film (blue squares), at selected *V*_GS_ and temperatures, respectively.

Comparing the sensitivities of the VO_2_ thin film and AlGaN/GaN HFET integrated cantilevers, we find that their maximum static and dynamic deflection sensitivities are very similar. In the linear dynamic regime, oscillation amplitudes of the cantilevers under study were expected to be the same, since they had the same dimensions and the same piezoactuator was utilized to excite them. Optimizing the sensitivity tuning parameters, namely, gate bias for AlGaN/GaN HFET, and temperature for VO_2_ thin film, led to the maximum sensitivity values of 0.5% (change in HFET *V*_DS_ at a constant *I*_DS_), and 0.35% (change in VO_2_ thin film *I*_DS_ at constant *V*_DS_ measured in linear regime applying 1 V to the piezoactuator), respectively. Both types of GaN microcantilevers also exhibited softening type nonlinearities at large deflection amplitudes. While the gate bias for the AlGaN/GaN HFET embedded microcantilever did not affect the intrinsic nonlinearities of the microcantilever at higher excitation amplitudes, increasing the operation temperature to modify the sensitivity of the VO_2_ thin film embedded microcantilever had a considerable influence over the nonlinear behavior, as discussed above. Moreover, the responses of the AlGaN/GaN HFET and VO_2_ thin film to 10 μm static downward bending support the experimental results observed in dynamic oscillation mode. Even though the AlGaN/GaN HFET and the VO_2_ thin film demonstrated opposite resistance change behaviors (decrease in *R*_DS_ for AlGaN/GaN HET, and increase in *R*_DS_ for the VO_2_ thin film) in response to the applied tensile stress, corresponding to a 10 μm step bending, their peak deflection transduction performances (16% change in *R*_DS_ for AlGaN/GaN HFET and 11% change in *R*_DS_ for VO_2_ thin film), at their optimized strain sensing conditions, are quite comparable.

## 4. Conclusions

In conclusion, we successfully compared the performances of piezotransistive AlGaN/GaN HFET and piezoresistive VO_2_ thin film based deflection transducers embedded on GaN microcantilevers in static and dynamic modes. Microcantilevers with similar nominal dimensions, when excited similarly with a piezoactuator, exhibited very similar maximum deflection sensitivities in the linear oscillation regime, which were tuned using gate bias and operation temperature, for AlGaN/GaN HFET and VO_2_ thin film transducers, respectively. In the linear dynamic regime, while the deflection sensitivity of the AlGaN/GaN HFET reached up to 0.5% change in *V*_DS_ at an appropriate gate bias of −2.5 V, the VO_2_ thin film deflection transducer demonstrated a maximum sensitivity of 0.36% change in *I*_DS_ around the MIT temperature of 65–75 °C. When operated in the non-linear regime, using higher excitation from the piezoactuator, a gradual reduction in sensitivities of the transducers was observed with both deflection transducers, while the VO_2_ embedded one showed variation drop frequencies at varying temperatures, due to changes in elastic modulus and dimensions. Comparable static deflection sensitivities were observed for the AlGaN/GaN HFET and the VO_2_ thin film when the microcantilevers were subjected to static bending. While a 10 μm downward tip bending resulted in a 16% change in *R*_DS_ of the AlGaN/GaN HFET at an optimal gate bias of −2.5 V, the maximum change in *R*_DS_ of the VO_2_ thin film, corresponding to the same downward step bending, was found to be 11%, at the critical MIT temperature of ~65 °C.

## References

[1] Ekinci K., Roukes M. (2005). Nanoelectromechanical Systems. Rev. Sci. Instrum..

[2] Qu H. (2016). CMOS MEMS Fabrication Technologies and Devices. Micromachines.

[3] Rugar D., Hansma P. (1990). Atomic Force Microscopy. Phys. Today.

[4] Yu X., Thaysen J., Hansen O., Boisen A. (2002). Optimization of sensitivity and noise in piezoresistive cantilevers. J. Appl. Phys..

[5] Boisen A., Dohn S., Keller S.S., Schmid S., Tenje M. (2011). Cantilever-like micromechanical sensors. Rep. Prog. Phys..

[6] Tortonese M., Barrett R., Quate C. (1993). Atomic resolution with an atomic force microscope using piezoresistive detection. Appl. Phys. Lett..

[7] Raiteri R., Grattarola M., Butt H., Skládal P. (2001). Micromechanical cantilever-based biosensors. Sens. Actuators B Chem..

[8] Waggoner P.S., Craighead H.G. (2007). Micro-and Nanomechanical sensors for environmental, chemical, and biological detection. Lab A Chip.

[9] Li M., Tang H.X., Roukes M.L. (2007). Ultra-sensitive NEMS-based cantilevers for sensing, scanned probe and very high-frequency applications. Nat. Nanotechnol..

[10] Shekhawat G., Tark S.H., Dravid V.P. (2006). MOSFET-embedded microcantilevers for measuring deflection in biomolecular sensors. Science.

[11] Zinoviev K., Dominguez C., Plaza J.A., Busto V.J.C., Lechuga L.M. (2006). A novel optical waveguide microcantilever sensor for the detection of nanomechanical forces. J. Lightwave Technol..

[12] Srinivasan K., Miao H., Rakher M.T., Davanco M., Aksyuk V. (2011). Optomechanical transduction of an integrated silicon cantilever probe using a microdisk resonator. Nano Lett..

[13] Gavartin E., Verlot P., Kippenberg T.J. (2012). A hybrid on-chip optomechanical transducer for ultrasensitive force measurements. Nat. Nanotechnol..

[14] Gurusamy J., Putrino G., Jeffery R.D., Silva K.D., Martyniuk M., Keating A., Faraone L. (2019). MEMS based hydrogen sensing with parts-per-billion resolution. Sens. Actuators B Chem..

[15] Tortonese M., Yamada H., Barrett R., Quate C. Atomic force microscopy using a piezoresistive cantilever. Proceedings of the TRANSDUCERS’91: 1991 International Conference on Solid-State Sensors and Actuators. Digest of Technical Papers.

[16] Sun Y., Thompson S., Nishida T. (2007). Physics of strain effects in semiconductors and metal-oxide-semiconductor field-effect transistors. J. Appl. Phys..

[17] Qazi M., DeRoller N., Talukdar A., Koley G. (2011). III-V nitride based piezoresistive microcantilever for sensing applications. Appl. Phys. Lett..

[18] Talukdar A., Khan M.F., Lee D., Kim S., Thundat T., Koley G. (2015). Piezotransistive transduction of femtoscale displacement for photoacoustic spectroscopy. Nat. Commun..

[19] Khan D., Bayram F., Gajula D., Talukdar A., Li H., Koley G. (2017). Plasmonic amplification of photoacoustic waves detected using piezotransistive gan microcantilevers. Appl. Phys. Lett..

[20] Bayram F., Khan D., Li H., Hossain M.M., Koley G. (2018). Piezotransistive GaN microcantilevers based surface work function measurements. Jpn. J. Appl. Phys..

[21] Talukdar A., Koley G. (2014). Impact of biasing conditions on displacement transduction by III-nitride microcantilevers. IEEE Electron Device Lett..

[22] Cabrera R., Merced E., Sepúlveda N., Fernández F.E. (2011). Dynamics of photothermally driven VO2-coated microcantilevers. J. Appl. Phys..

[23] Cabrera R., Merced E., Sepúlveda N. (2013). Performance of electro-thermally driven ${\rm VO} _ {2} $-based MEMS actuators. J. Microelectromechanics Syst..

[24] Manrique-Juarez M.D., Rat S., Salmon L., Molnár G., Quintero C.M., Nicu L., Shepherd H.J., Bousseksou A. (2016). Switchable molecule-based materials for micro-and nanoscale actuating applications: Achievements and prospects. Coord. Chem. Rev..

[25] Rúa A., Cabrera R., Coy H., Merced E., Sepúlveda N., Fernández F.E. (2012). Phase transition behavior in microcantilevers coated with M1-Phase VO2 and M2-Phase VO2: Cr thin films. J. Appl. Phys..

[26] Gajula D., Bayram F., Jahangir I., Khan D., Koley G. Dynamic response of VO 2 mesa based GaN microcantilevers for sensing applications. Proceedings of the 2019 IEEE Sensors.

[27] Stefanovich G., Pergament A., Stefanovich D. (2000). Electrical switching and mott transition in VO2. J. Phys. Condens. Matter.

[28] Kalcheim Y., Camjayi A., del Valle J., Salev P., Rozenberg M., Schuller I.K. (2020). Non-Thermal resistive switching in mott insulator nanowires. Nat. Commun..

[29] Fan L., Chen S., Luo Z., Liu Q., Wu Y., Song L., Ji D., Wang P., Chu W., Gao C. (2014). Strain Dynamics of Ultrathin VO2 Film Grown on TiO2 (001) and the Associated Phase Transition Modulation. Nano Lett..

[30] Cao J., Ertekin E., Srinivasan V., Fan W., Huang S., Zheng H., Yim J., Khanal D., Ogletree D., Grossman J. (2009). Strain engineering and one-dimensional organization of metal–insulator domains in single-crystal vanadium dioxide beams. Nat. Nanotechnol..

[31] Singh R., Khan D., Gajula D., Bayram F., Koley G. Synthesis and characterization of VO 2 on III nitride thin films using low pressure chemical vapor deposition for sensing applications. Proceedings of the 2018 IEEE 13th Nanotechnology Materials and Devices Conference (NMDC).

[32] Azad S., Singh R., Munna M., Bayram F., Khan D., Li H., Koley G. Investigation of VO 2 Thin Film Grown on III-Nitride Epitaxial Layer. Proceedings of the 2020 IEEE 20th International Conference on Nanotechnology (IEEE-NANO).

[33] Khan D., Li H., Bayram F., Gajula D., Koley G. (2020). Photoacoustic detection of H2 and NH3 using plasmonic signal enhancement in GaN microcantilevers. Micromachines.

[34] Bayram F., Gajula D., Khan D., Gorman S., Koley G. (2019). Nonlinearity in piezotransistive GaN microcantilevers. J. Micromechanics Microengineering.

[35] Okimura K., Kubo N. (2005). Preparation of VO2 films with metal–insulator transition on sapphire and silicon substrates by inductively coupled plasma-assisted sputtering. Jpn. J. Appl. Phys..

[36] Yu S., Wang S., Lu M., Zuo L. (2017). A metal-insulator transition study of VO2 thin films grown on sapphire substrates. J. Appl. Phys..

[37] Mertens J., Finot E., Thundat T., Fabre A., Nadal M., Eyraud V., Bourillot E. (2003). Effects of temperature and pressure on microcantilever resonance response. Ultramicroscopy.

[38] Kacem N., Arcamone J., Perez-Murano F., Hentz S. (2010). Dynamic range enhancement of nonlinear nanomechanical resonant cantilevers for highly sensitive NEMS Gas/Mass sensor applications. J. Micromechanics Microengineering.

[39] Rhoads J.F., Shaw S.W., Turner K.L. (2010). Nonlinear dynamics and its applications in micro-and nanoresonators. J. Dyn. Syst. Meas. Control..

[40] Lifshitz R., Cross M. (2008). Nonlinear dynamics of nanomechanical and micromechanical resonators. Rev. Nonlinear Dyn. Complex..

[41] Imboden M., Williams O., Mohanty P. (2013). Nonlinear dissipation in diamond nanoelectromechanical resonators. Appl. Phys. Lett..

[42] Imboden M., Mohanty P. (2014). Dissipation in nanoelectromechanical systems. Phys. Rep..

[43] Hu B., Ding Y., Chen W., Kulkarni D., Shen Y., Tsukruk V.V., Wang Z.L. (2010). External-strain induced insulating phase transition in VO2 nanobeam and its application as flexible strain sensor. Adv. Mater..

